# Supplementary material for: An open-source framework for large-scale, flexible evaluation of biomedical text mining systems
Source: J Biomed Discov Collab. 2008 Jan 29;3:1. doi: 10.1186/1747-5333-3-1 (PMC2276192; doi:10.1186/1747-5333-3-1)
Supplement: Additional file 1 — Evaluation platform source code. The additional file contains the software evaluation framework discussed in this paper. The most current release can be downloaded from SourceForge.net [1]. See the accompanying README file for instructions on installation and use. Also included in the distribution is a collection of UIMA wrappers for some commonly used BioNLP tools and annotated corpora. [file 1747-5333-3-1-S1.zip › bionlp_uima_repository-1.0.1/docs/api/edu/uchsc/ccp/util/nlp/annotation/comparison/SharedStartOrEndSpanComparator.html]

SharedStartOrEndSpanComparator (Center for Computational Pharmacology Util/UIMA API)


|  |  |  |
| --- | --- | --- |
| **Overview** \| **Package** \| **Class** \| **Tree** \| **Deprecated** \| **Index** \| **Help** | |  |
| **PREV CLASS**   **NEXT CLASS** | **FRAMES**    **NO FRAMES**     **All Classes** |  |
| SUMMARY: NESTED \| FIELD \| CONSTR \| METHOD | DETAIL: FIELD \| CONSTR \| METHOD |  |


---


## edu.uchsc.ccp.util.nlp.annotation.comparison Class SharedStartOrEndSpanComparator

```
java.lang.Object
  edu.uchsc.ccp.util.nlp.annotation.comparison.SpanComparator
      edu.uchsc.ccp.util.nlp.annotation.comparison.SharedStartOrEndSpanComparator
```

**All Implemented Interfaces:**: Comparator<Span>

---

``` public class SharedStartOrEndSpanComparator extends SpanComparator ```

For "shared start or end" comparison, the spans are required to have the same start or end indexes in order to return 0. If they do not share the
same span start or end, then if the span start for span1 is prior to the span start for span2, then -1 is returned, 1 otherwise.

**Author:**
:   williamb

---

**Constructor Summary**

`SharedStartOrEndSpanComparator()`


| **Method Summary** | |
| --- | --- |
| `int` | `compare(List<Span> spanList1, List<Span> spanList2)`             Compare two lists of spans using the shares-span-start-or-end match criteria. |
| `int` | `compare(Span span1, Span span2)`             Compare two spans using the shares-span-start-or-end match criteria. |

**Methods inherited from class edu.uchsc.ccp.util.nlp.annotation.comparison.SpanComparator**

`matches, matches, overlaps, overlaps, sharesSpanEnd, sharesSpanEnd, sharesSpanStart, sharesSpanStart, sharesSpanStartOrEnd, sharesSpanStartOrEnd`

**Methods inherited from class java.lang.Object**

`clone, equals, finalize, getClass, hashCode, notify, notifyAll, toString, wait, wait, wait`

**Methods inherited from interface java.util.Comparator**

`equals`

**Constructor Detail**

### SharedStartOrEndSpanComparator

```
public SharedStartOrEndSpanComparator()
```


**Method Detail**

### compare

```
public int compare(Span span1,
                   Span span2)
```

:   Compare two spans using the shares-span-start-or-end match criteria.

    :   **Specified by:**: `compare` in interface `Comparator<Span>` **Specified by:**: `compare` in class `SpanComparator`

---


### compare

```
public int compare(List<Span> spanList1,
                   List<Span> spanList2)
```

:   Compare two lists of spans using the shares-span-start-or-end match criteria.

    :   **Specified by:**: `compare` in class `SpanComparator`
    :   **Parameters:**: `spanList1` -: `spanList2` - **Returns:**


---


|  |  |  |
| --- | --- | --- |
| **Overview** \| **Package** \| **Class** \| **Tree** \| **Deprecated** \| **Index** \| **Help** | |  |
| **PREV CLASS**   **NEXT CLASS** | **FRAMES**    **NO FRAMES**     **All Classes** |  |
| SUMMARY: NESTED \| FIELD \| CONSTR \| METHOD | DETAIL: FIELD \| CONSTR \| METHOD |  |


---
